# Auroral molecular-emission effects on the atomic oxygen line at 777.4 nm

**DOI:** 10.1186/s40623-018-0936-z

**Published:** 2018-10-16

**Authors:** Shin-ichiro Oyama, Takuo T. Tsuda, Keisuke Hosokawa, Yasunobu Ogawa, Yoshizumi Miyoshi, Satoshi Kurita, Antti E. Kero, Ryoichi Fujii, Yoshimasa Tanaka, Akira Mizuno, Tetsuya Kawabata, Björn Gustavsson, Thomas Leyser

**Affiliations:** 1 ISEE Nagoya University, F3-3 Furo Chikusa, Nagoya, Aichi 464-8601 Japan; 2 Ionospheric Physics Research Unit, University of Oulu, Pentti Kaiteran katu 1, Linnanmaa, 90014 Oulu, Finland; 3 National Institute of Polar Research, 10-3, Midori-cho, Tachikawa-shi, Tokyo 190-8518 Japan; 4 The University of Electro-Communications, 1-5-1 Chofugaoka, Chofu, Tokyo 182-8585 Japan; 5 Sodankylä Geophysical Observatory, Tähteläntie 62, 99600 Sodankylä, Finland; 6 Research Organization of Information and Systems, Toranomon 4-3-13, Minato-ku, Tokyo, 105-0001 Japan; 7 University of Tromsø, Norway, Hansine Hansens veg 18, 9019 Tromsø, Norway; 8 Swedish Institute of Space Physics, Box 537, 751 21 Uppsala, Sweden

**Keywords:** Aurora, Ionosphere, Magnetosphere, Spectrograph, EISCAT

## Abstract

One of the representative auroral emission lines that radiates from F-region heights and is measurable on the ground is the 777.4 nm line from excited atomic oxygen. This line has been adopted, along with another E-region emission line, for example 427.8 nm, to estimate the mean energy and total energy flux of precipitating auroral electrons. The influence of emissions from part of the molecular nitrogen band, which mainly radiate from E-region heights, should be carefully evaluated because it might overlap the 777.4 nm atomic oxygen line in the spectrum. We performed statistical analysis of auroral spectrograph measurements that were obtained during the winter of 2016–2017 in Tromsø, Norway, to derive the ratio of the intensity of the 777.4 nm atomic oxygen line to that of the net measurement through a typically used optical filter with a full width at half maximum of a few nm. The ratio had a negative trend against geomagnetic activity, with a primary distribution of 0.5–0.7 and a minimum value of 0.3 for the most active auroral condition in this study. This result suggests that the 30–50% emission intensities measured through the optical filter may be from the molecular nitrogen band. 
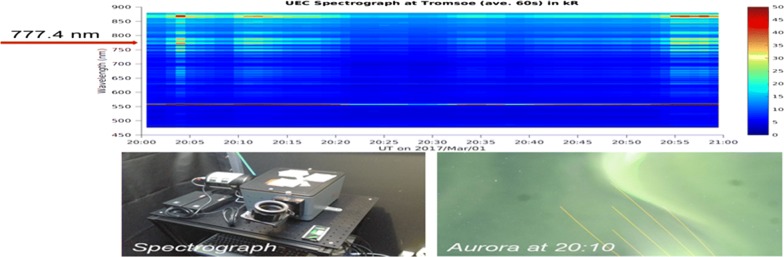

## Introduction

The energy flux of auroral electrons is one of the fundamental parameters that are used to elucidate precipitation generation mechanisms. Methods that are applicable to ground-based measurements for energy-flux estimation have been developed for more than three decades. One is the application of optical measurements for a set of prompt emitters that predominantly originate in the E and F regions. The expectation of this approach is that E- and F-region emissions may contain information about hard and soft energy precipitations, respectively, and the ratio between the two intensities may be related to precipitating-electron energy and flux. For example, Ono (1993) chose 427.8 nm for the E-region emission line and tested wavelengths of 777.4 nm and 844.6 nm for the F-region emission lines. Both F-region lines are radiated from excited atomic oxygen. These lines are also studied by other groups via measurements (Dahlgren et al. 2008; Lanchester et al. 2009; Dahlgren et al. 2011; Lanchester and Gustavsson 2012; Tuttle et al. 2014) and simulations, such as the latest version of the GLobal airglOW (GLOW) model (https://zenodo.org/record/241139) and a code of the UK group (see Ashrafi et al. (2009) and Lanchester et al. (2009)).

In September 2017, we began the operation of two EMCCD cameras in Tromsø, Norway, at a sampling rate of 10 Hz. One of the main scientific objectives is horizontal two-dimensional energy-flux estimation during the appearance of pulsating aurora by applying the method that is mentioned above. The estimated energy flux will be compared with measurements from the ARASE satellite and the European Incoherent Scatter (EISCAT) radar. One of the EMCCD cameras uses an optical filter that is centered near 427.8 nm to measure the E-region emission line. For the F-region emission line, we decided to measure emission intensities at 844.6 nm instead of 777.4 nm based on results from this study.

The 777.4 nm emission that originates in the F region is caused by radiative recombination of O^+^, which leaves atomic oxygen in an excited state (O^+^(^4^S) + e^−^ → O*) (Tinsley et al. 1973; Makela et al. 2001, Makela and Kelley 2003). The excited atomic oxygen (O*) cascades to the ground state while radiating light at a wavelength of 777.4 nm. Dissociative excitation of molecular oxygen can also emit light at 777.4 nm, which comes from the E region. Hecht et al. (1985) obtained a high-resolution measurement of the atomic oxygen line at 777.4 nm [hereinafter written as OI (777.4)] with a Fabry–Perot interferometer, which consisted of two components: a narrow spectral portion, which is due to the excitation of atomic oxygen via direct electron impact, and a broad portion, which is due to the dissociative excitation of molecular oxygen via electron impact. However, the contribution of the latter component tends to be small, constituting no more than 5% of the OI (777.4) emission intensity (Christensen et al. 1978). The volume emission rates of the OI (777.4) in the F region exceed those in the E region by a factor of 2 or more [see Figure 1 of Lanchester et al. (2009)]. This suggests that the majority of the OI (777.4) emission intensity that is measured on the ground originated in the F region rather than the E region.

However, a matter of concern in the application of OI (777.4) for energy-flux estimation has been the influence of the first positive band of molecular nitrogen (hereinafter written as N2 1PG), which appears at 765–780 nm in the spectrum. This is an emission that comes predominantly from the E region; it is thus more sensitive to the harder energies of the precipitating electrons than expected in the method to be employed. For example, according to the spectrum that was reported by Jones (1974; Figure 4.6 in the book), the longer-wavelength part of N2 1PG (2,0) band overlaps the OI (777.4) line, although there is a moderately clear valley in the spectrum between N2 1PG (2,0) and OI (777.4). At high latitudes, particularly in the auroral ionosphere, particle precipitations likely consist of both soft and hard electrons at the same time. It is thus essential to evaluate the influence of N2 1PG (2,0) on the measurement at 777.4 nm. In this study, we analyzed data from a spectrograph that was deployed in Tromsø in October 2016 to separate the OI (777.4) part of the measurement. The method will be presented in “Methodology for extracting the OI (777.4) line” section. “Results” section describes the events and presents the statistical results. The results and conclusions are discussed in “Discussion and conclusions” section.

## Methodology for extracting the OI (777.4) line

A compact spectrograph was developed for measuring natural and artificial atmospheric emissions at the University of Electro-Communications, Japan. The spectrograph was deployed at the European Incoherent Scatter (EISCAT) radar site in Tromsø, Norway (69.6°N, 19.2°E) after careful calibration at the National Institute of Polar Research and operation began on October 4, 2016. The spectrograph was tuned for measuring visible emission intensities (mainly at wavelengths of 480–880 nm) by grating collimated light (300 G/mm) that had passed through a slit (100 μm width). The wavelength resolution is approximately 1.6 nm with a sampling interval of approximately 0.4 nm. The aperture (i.e., F-number) was approximately 4. The rectangular field-of-view (FOV; 0.03° in elevation and 2° in azimuth) was pointed at the local magnetic zenith. The data-sampling interval was 1 s, which included an exposure time of 0.7 s. More than 8,000,000 measurements had been obtained by April 15, 2017.

The atomic oxygen line at 777.4 nm, which is denoted as OI (777.4), is embedded in the N2 1PG (2,0) band in the spectrum. In exploring the possibility of capturing signatures of soft-electron precipitation from the OI (777.4) line, it is essential to elucidate the effects of the N2 1PG (2,0) band, as discussed in “Introduction” section. In many cases, electron precipitation energies are likely to be distributed over a wide range such as 0.1–100 keV. Since neutral particles at both E- and F-region heights are excited simultaneously by these precipitating electrons, passive optical instruments that are deployed on the ground record integrated emissions that are radiated from OI (777.4) and N2 1PG (2,0), even if a narrow-band optical filter is mounted on the instrument. However, in some cases, the energy distribution is moderately biased toward high energies, thereby resulting in an electron density peak at a relatively low altitude of the ionosphere and little increase in the F-region density. In this case, the emission intensity of N2 1PG (2,0) may be high enough to ignore that of OI (777.4) and we can assume that the spectrum near 777.4 nm is representative of quasi-pure emission from N2 1PG (2,0). In contrast, the spectrum that is principally attributed to soft-electron precipitation can be adopted as representative of quasi-pure emission from OI (777.4). The type of electron precipitation can be identified by carefully examining the height profile of the EISCAT-measured electron density.

Surveying all available measurements from the spectrograph and EISCAT radar from October 9, 2016, to April 15, 2017, we found an acceptable spectrum from a simultaneous experiment that was conducted on March 2, 2017. The spectrum will be presented in the next section. The event meets the criteria of a single E-region peak and an unremarkably small increase in the F-region density. The spectrum is thus regarded as a measurement that is principally attributed to E-region emissions with less influence from the F region (hereinafter called the “reference spectrum”). Adequate events that were associated with soft-electron precipitation or a single F-region density peak were not found for the period that was selected in this study.

Measured spectra can be modeled as weighted sums of the reference spectrum (N2 1PG) and OI (777.4). In other words, emission intensities of OI (777.4) can be extracted by subtracting the weighted reference spectrum from the measured spectra; the background noise should be removed in advance. After converting to the absolute emission intensity in Rayleigh, the background noise for each spectrum is defined as the mean emission intensity at 760.5–763.5 nm. This wavelength range is located in a valley between the spectral peaks of N2 1PG (3,1) and (2,0) (Jones 1974), and the measured spectra have the smallest statistical standard deviation in the wavelength range of 750–800 nm. Since N2 1PG (2,0) has a clear peak at 770 nm, in this study, the weighting coefficients are chosen to match the emission intensity of the reference spectrum at 770 nm to those that were measured for individual spectra.

While we believe that this method is practical, there are a few cautions when applying it to real measurements: First, the measured spectrum may include the OI (777.4) line, even for a single E-region density peak, because it can be emitted from the E region (see “Introduction” section). Second, the F-region ionization rate and the OI (777.4) emission at F-region heights never completely disappear, even if the stopping height of a precipitating mono-energetic electron is in the E region. Third, the lower-energy part of the precipitating electron spectrum can cause the OI (777.4) emission in the F region even when the electron density has a single peak in the E region. All three scenarios will cause ambiguity in the method mentioned that is discussed above and will result in overestimation at the OI (777.4) wavelength in the reference spectrum. This effect will be discussed in “Discussion and conclusions” section.

For extracting the OI (777.4) line from the measured spectra, measurement during a single F-region density peak is more suitable than during a single E-region density peak. However, the latter was adopted in this study. This is because soft-electron precipitations do not cause ionization at E-region heights or N2 1PG band emissions. The OI (777.4) intensity can be derived by simply adjusting the peak of the reference spectrum to match the measured spectrum via application of a multiplicative factor. However, such precipitation scarcely occurs in the auroral oval, although it might be observed in the polar cap and the cusp regions.

## Results

**Fig. 1:**
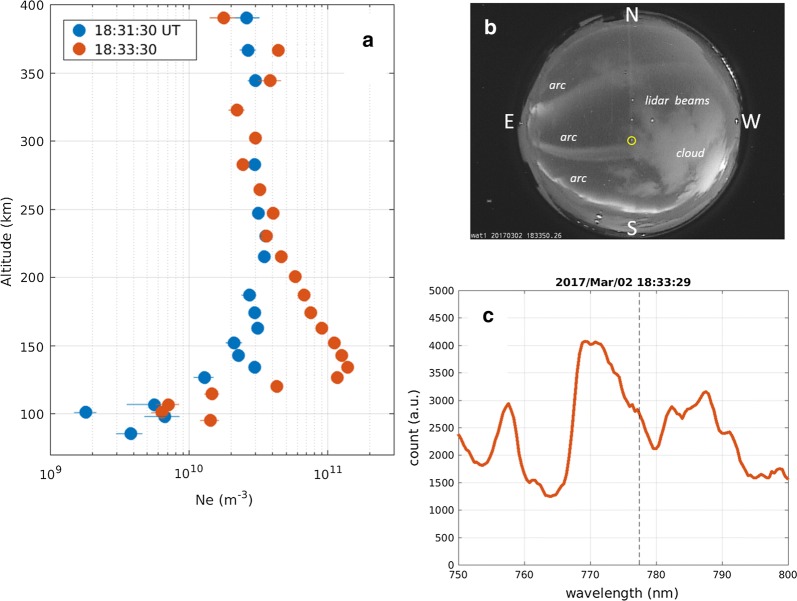
**a** Height profiles of the EISCAT-measured electron density at 18:31:30 UT (blue) and 18:33:30 UT (orange) on March 2, 2017. Measurement uncertainty of ± 1*σ* is marked by a horizontal bar in each color. **b** An image of the Tromsø all-sky camera, which was taken at 18:33:50 UT. The direction of the EISCAT radar and spectrograph measurements is marked by a yellow circle. Five faint lines from the northern edge represent contaminations of a sodium lidar. **c** Spectrum measured at 18:33:29 UT on March 2, 2017, at wavelength of 750–800 nm. A vertical dashed line is marked at 777.4 nm

Figure 1a shows two height profiles of the electron density, which was measured with the EISCAT UHF radar on March 2, 2017; the device was fixed to look along the geomagnetic field line. Measurement uncertainties (± 1*σ*) are overlaid on each measurement with horizontal lines. Comparing with the electron density at 18:31:30 UT (the time stamp is at the center of a 1-min integration; blue dots in Fig. 1a), the measurement at 18:33:30 UT (orange dots) is characterized by a single E-region peak at 130 km and a smaller increase in the F-region density (above 210 km). Thus, the spectrograph measurement at 18:33 UT can be adopted as the reference spectrum of the principal N2 1PG band.

These EISCAT measurements were made near a zonally elongated narrow arc. Figure 1b presents a snapshot that was taken with a collocated all-sky camera at 18:33:50 UT. The direction of the EISCAT radar beam and the spectrograph is marked by a yellow circle. Five faint lines from the northern edge are contaminations that were emitted from a collocated sodium lidar. While the western half of the arc was behind a cloud patch, the effects of water vapor absorption are not significant near 777.4 nm (Noël et al. 2000). Three arcs that were observed in the sky moved equatorward without notable change in shape or intermittence. By examining successive images, we concluded that at 18:31–18:32 UT, when the electron density was moderately low in the E region (blue dots in Fig. 1a), the EISCAT measurement was initially made relatively equatorward, on the side away from the arc, and subsequently moved inside or at the edge of the arc at 18:33–18:34 UT, when the E-region density had a peak (orange dots in Fig. 1a).

Figure 1c shows the spectrum from 750 to 800 nm that was obtained at 18:33–34 UT with the spectrograph (directed along B). The N2 1PG (2,0) band is clearly observed with a peak approximately 770 nm, as expected from the E-region peak of the electron-density height profile at 18:33:30 UT (orange dots in Fig. 1a). The OI (777.4) line is not clearly identified in the spectrum because its emission intensity is probably considerably smaller than the intensity of N2 1PG (2,0). However, a bump that is observed approximately 777.4 nm might be a portion of the OI (777.4) line. While we do not claim that the spectrum that is shown in Fig. 1c does not contain any OI (777.4) emissions, its contribution to the spectrum is small enough to ignore.

**Fig. 2:**
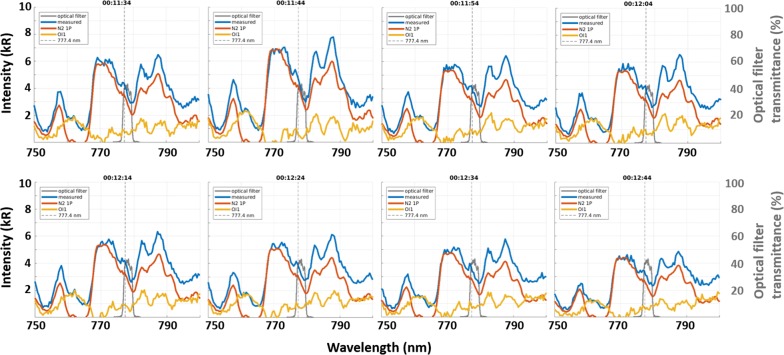
Eight spectra that were measured with the spectrograph at wavelengths of 750–800 nm from 00:11:34 to 00:12:44 UT on March 2, 2017 (blue). A vertical dashed line is marked at 777.4 nm. The orange and yellow curves are explained in the text. The transmittance of the optical filter that is mounted on the EMCCD camera in Tromsø is represented in by a gray curve

Figure 2 consists of spectra that were obtained every 10 s from 00:11:34 to 00:12:44 UT on March 2, 2017 (in blue). The figure contains data that were obtained on same date as the measurement of the reference spectrum but at different times and both soft- and hard-electron precipitations are included. The reference spectrum, which is shown in Fig. 1c, is drawn as an orange curve after fitting it to individual measurements via the method that was introduced in “Methodology for extracting the OI (777.4) line” section. The spectrum of the optical filter that was employed with the EMCCD camera is shown as a gray curve. The center wavelength of the optical filter deviates from 777.4 nm by 0.8 nm toward longer wavelength to reduce contamination from N2 1PG (2,0). The differences between the measurements and the reference spectrum, which are shown as yellow curves, have been calculated. The OI (777.4) line should appear in a narrow wavelength range of the yellow curve near 777.4 nm if it appears at all. For example, one may be able to see a moderately sharp peak near 777.4 nm (but not at exactly 777.4 nm due to the instrumental resolution of 1.6 nm) at 00:12:24 and 00:12:34 UT in both the measured (blue) and the residual (yellow) spectra. However, the N2 1PG bands obviously overlap the OI (777.4) line. While all available measurements have been checked, these two spectra may be examples of the OI (777.4) line that is observed via the spectrograph.

**Fig. 3:**
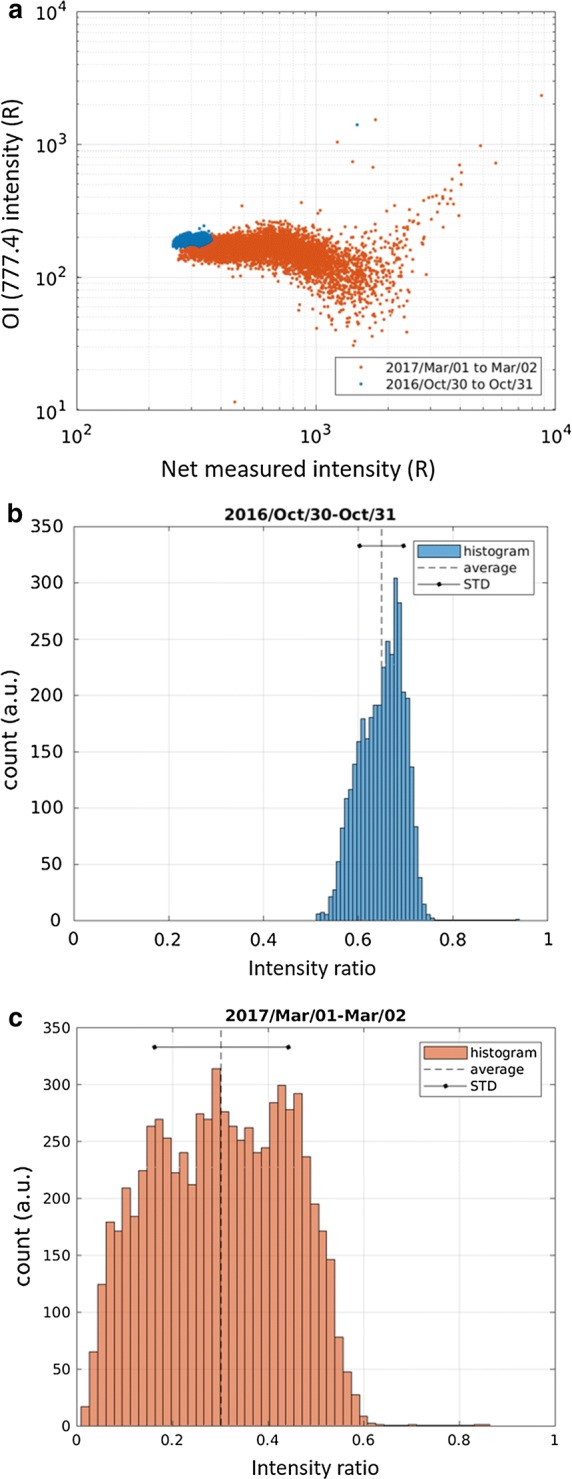
**a** Relationship between estimated emission intensities of OI (777.4) (*I*_OI_) and the value to be measured through the optical filter that is mounted on the EMCCD camera (*I*_m_) on the nights of (blue) October 30–31, 2016 and (orange) March 1–2, 2017. **b** Histogram of the emission intensity ratio (*I*_OI_/*I*_m_) on night of October 30–31, 2016. The statistical average and standard deviation are marked by a black dash and a solid line with dots, respectively. **c** Same as panel **b**, but for data from the night of March 1–2, 2017

In measurements with the camera deployed at the site, light passes through the optical filter (see the gray curve in Fig. 2). To obtain values under realistic conditions, spectrograph measurements are integrated at wavelengths in the optical filter spectrum. A weighting function that is based on the transmittance is adopted for the calculation. The N2 1PG band, the OI (777.4) line, and the net value (orange, yellow, and blue curves in Fig. 2, respectively; hereinafter written as $I_{{{\text{N}}_{2} }}$, *I*_OI_, and *I*_m_, respectively) are separately derived for each measurement. As an example, Fig. 3a shows relationships between *I*_m_ and *I*_OI_ on two nights: October 30–31, 2016 (blue) and March 1–2, 2017 (orange). The geomagnetic activity, which is defined as the mean Ap value over the period of each nightly measurement, was 9 and 60, respectively; hence, it is assumed that the corresponding periods are moderately and highly active periods, respectively. Histograms of the intensity ratio (*I*_OI_/*I*_m_), which are shown in Fig. 3b, c, clearly show a difference in the standard deviation (smaller for the former case than for the latter case). Of particular interest is the mean value of the intensity ratio. The mean value for October 30–31, 2016 (Fig. 3b) is obviously larger than that for March 1–2, 2017 (Fig. 3c). This result suggests that radiation from the N2 1PG band constitutes a more significant contamination for the latter case than for the former case.

**Fig. 4:**
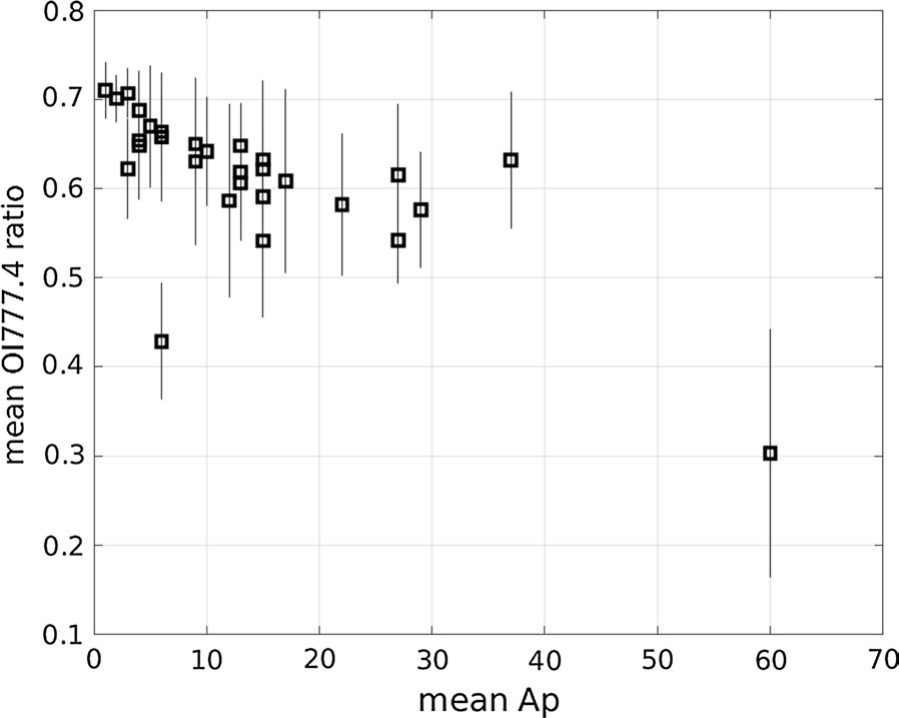
Geomagnetic activity dependency of the mean OI (777.4) ratio, which is derived from all available data that were obtained from October 9, 2016 to April 15, 2017. The vertical bar is the statistical standard deviation (± 1*σ*)

The two events that are shown in Fig. 3 demonstrate that level of influence of the N2 1PG band on OI (777.4) has a positive dependence on the geomagnetic activity. We performed a statistical analysis of all available data that were obtained from October 6, 2016, to April 15, 2017, to determine whether the trend that is suggested in Fig. 3 is a representative signature. The method that was employed to produce Fig. 3 was applied to the data set and mean values for 30 nights are plotted as a function of the mean Ap value to produce Fig. 4. Statistical standard deviations (± 1*σ*) are indicated by vertical bars. Mean values of the intensity ratio (*I*_OI_/*I*_m_) tend to decrease with geomagnetic activity. A different but equivalent interpretation is that contamination that results from enhancements of emission intensity that are attributed to the N2 1PG band tends to increase with the geomagnetic activity. The intensity ratio falls below approximately 0.7 for all of the cases that are analyzed in this study, and in the most extreme case, the ratio reaches approximately 0.3 (as shown in Fig. 3c). A negative trend of the intensity ratio can be identified even if the extreme case (Ap = 60) is ignored. That is, the intensity ratio (*I*_OI_/*I*_m_) can be anywhere in the range 0.3–0.7.

## Discussion and conclusions

In this study, we analyzed spectrograph data that were obtained during auroral appearances at Tromsø, Norway, from October 9, 2016, to April 15, 2017, without taking into account the local time or the morphology. A method for excluding the emissions of the N2 1PG band near 777.4 nm was developed to evaluate the ratio of the OI (777.4) intensity to that measured through the optical filter with a full width at half maximum of a few nm. Since emissions from both OI (777.4) and the N2 1PG band comprise the measured intensity, a higher ratio suggests a lower level of content of the N2 1PG band. Statistical analysis demonstrated that the intensity ratio (*I*_OI_/*I*_m_) tends to decrease with the enhancement of geomagnetic activity. This is due to a positive correlation of hard-electron precipitation flux with geomagnetic activity. While the soft-electron precipitation flux may also increase with geomagnetic activity, ionization and excitation of neutral particles are more significant at E-region heights than at F-region heights. Therefore, the emission intensity of the N2 1PG band increases more remarkably than that of OI (777.4).

The statistical analysis demonstrates that the ratio has a primary distribution of 0.5–0.7 and a minimum value of 0.3 for the most active auroral scenario in this study. However, the ratio might be slightly larger than the derived ratio because of ambiguity in estimating the reference spectrum. The reference spectrum does not consist purely of the N2 1PG band due to plausible contaminations from the OI (777.4) line, as discussed in “Methodology for extracting the OI (777.4) line” section. The estimated OI (777.4) line as the residual may be larger than the line that was observed in this study, but not sufficiently large to change the conclusion. Otherwise, the OI (777.4) line would have been clearly observed in the measurements without post-analyses.

We might have held an unverifiable impression of the OI (777.4) line that was measured at auroral latitudes. According to a spectrograph that was presented by Jones (1974), the OI (777.4) line is moderately separated from the neighboring N2 1PG bands by a gap in wavelength. However, at least in the spectrograph measurements that we analyzed in this study, such a clear wavelength gap was never observed. While the wavelength resolution was 1.6 nm with a 0.4 nm sampling interval in the measurement, that is not the primary reason for the absence of a clear gap, the gap is absent due to intrusion of the N2 1PG (2,0) band to a longer wavelength than 777.4 nm with increasing the emission intensity. It is difficult to avoid contamination from the N2 1PG band as we are employing normal optical filters that are made of thin films deposited on a glass plate. Application of a narrow interference optical filter (e.g., full width at half maximum of ~1 nm) may reduce the effects (Dahlgren et al. 2015), but it is difficult to remove them completely. The main conclusion of this study is that the OI (777.4) line is not appropriate for measuring F-region emissions for energy estimation at auroral latitudes. Other atomic oxygen lines, such as 844.6 nm, may be more reliable.

## Abbreviations

GLOW: Global airglow
UK: United Kingdom
EMCCD: electron multiplying charge coupled device
EISCAT: European Incoherent Scatter
1PG: first positive
FOV: field-of-view

## References

[CR1] Ashrafi M, Lanchester BS, Lummerzheim D, Ivchenko N, Jokiaho O (2009) Modelling of N2 1P emission rates in aurora using various cross sections for excitation. Ann Geophys 27:2545–2553. http://www.ann-geophys.net/27/2545/2009/

[CR2] Christensen AB, Rees MH, Romick GJ, Sivjee GG (1978). OI (7774 Å) and OI (8446 Å) emission in aurora. J Geophys Res.

[CR3] Dahlgren H, Ivchenko N, Lanchester BS, Sullivan J, Whiter D, Marklund G, Strømme A (2008) Using spectral characteristics to interpret auroral imaging in the 731.9 nm O^+^ line. Ann Geophys. 10.5194/angeo-26-1905-2008

[CR4] Dahlgren H, Gustavsson B, Lanchester BS, Ivchenko N, Brändström U, Whiter DK, Sergienko T, Sandahl I, Marklund G (2011) Energy and flux variations across thin auroral arcs. Ann Geophys. 10.5194/angeo-29-1699-2011

[CR5] Dahlgren H, Lanchester BS, Ivchenko N (2015). Coexisting structures from high- and low-energy precipitation in fine-scale aurora. Geophys Res Lett.

[CR6] Hecht JH, Christensen AB, Pranke JB (1985). High-resolution auroral observations of the OI(7774) and OI(8446) multiplets. Geophys Res Lett.

[CR7] Jones Alister Vallance (1974). Aurora.

[CR8] Lanchester Betty, Gustavsson Björn (2012). Imaging of Aurora to Estimate the Energy and Flux of Electron Precipitation. Auroral Phenomenology and Magnetospheric Processes: Earth and Other Planets.

[CR9] Lanchester BS, Ashrafi M, Ivchenko N (2009) Simultaneous imaging of aurora on small scale in OI (777.4 nm) and N21P to estimate energy and flux of precipitation. Ann Geophys 10.5194/angeo-27-2881-2009

[CR10] Makela JJ, Kelley MC (2003). Field-aligned 777.4-nm composite airglow images of equatorial plasma depletions. Geophys Res Lett.

[CR11] Makela JJ, Kelley MC, Gonzalez SA, Aponte N, McCoy RP (2001). Ionospheric topography maps using multiple-wavelength all-sky images. J Geophys Res.

[CR12] Noël S, Bovensmann H, Burrows JP (2000) Water vapour retrieval from GOME data including cloudy scenes. In: Proceedings of the ENVISAT/ERS symposium, Gothenburg

[CR13] Ono T (1993). Derivation of energy parameters of precipitating auroral electrons by using the intensity ratios of auroral emissions. J Geomag Geoelec.

[CR14] Tinsley BA, Christensen AB, Bittencourt J, Gouveia H, Angreji PD, Takahashi H (1973). Excitation of oxygen permitted line emissions in the tropical nightglow. J Geophys Res.

[CR15] Tuttle S, Gustavsson B, Lanchester B (2014). Temporal and spatial evolution of auroral electron energy spectra in a region surrounding the magnetic zenith. J Geophys Res Space Phys.

